# Computation of condition-dependent proteome allocation reveals variability in the macro and micro nutrient requirements for growth

**DOI:** 10.1371/journal.pcbi.1007817

**Published:** 2021-06-23

**Authors:** Colton J. Lloyd, Jonathan Monk, Laurence Yang, Ali Ebrahim, Bernhard O. Palsson

**Affiliations:** 1 Department of Bioengineering, University of California, San Diego, La Jolla, California, United States of America; 2 Novo Nordisk Foundation Center for Biosustainability, Technical University of Denmark, Lyngby, Denmark; 3 Department of Pediatrics, University of California, San Diego, La Jolla, California, United States of America; Ecole Polytechnique Fédérale de Lausanne, SWITZERLAND

## Abstract

Sustaining a robust metabolic network requires a balanced and fully functioning proteome. In addition to amino acids, many enzymes require cofactors (coenzymes and engrafted prosthetic groups) to function properly. Extensively validated resource allocation models, such as genome-scale models of metabolism and gene expression (ME-models), have the ability to compute an optimal proteome composition underlying a metabolic phenotype, including the provision of all required cofactors. Here we apply the ME-model for *Escherichia coli* K-12 MG1655 to computationally examine how environmental conditions change the proteome and its accompanying cofactor usage. We found that: (1) The cofactor requirements computed by the ME-model mostly agree with the standard biomass objective function used in models of metabolism alone (M-models); (2) ME-model computations reveal non-intuitive variability in cofactor use under different growth conditions; (3) An analysis of ME-model predicted protein use in aerobic and anaerobic conditions suggests an enrichment in the use of peroxyl scavenging acids in the proteins used to sustain aerobic growth; (4) The ME-model could describe how limitation in key protein components affect the metabolic state of *E*. *coli*. Genome-scale models have thus reached a level of sophistication where they reveal intricate properties of functional proteomes and how they support different *E*. *coli* lifestyles.

## Author summary

*Escherichia coli* is capable of growing in many environments, each of which requires a different collection of enzymes to metabolize the nutrients within that environment. Each individual enzyme requires its own set of amino acids and oftentimes cofactors, which are accessory molecules essential for the enzyme to function. Thus, the composition of the micronutrients (amino acids, cofactors, etc.) within a cell will differ depending on its metabolic needs. The presented work is the first effort to employ metabolic models to probe the connection between *E*. *coli*’s diverse growth environments and its biomass composition. We first show how differences in model-predicted enzyme use for aerobic or anaerobic growth results in distinct amino acid and cofactor usage. Alternatively, we show that the metabolic models can predict how modifying the cell’s biomass composition will affect growth. For example, by modeling the exposure of *E*. *coli* to trimethoprim or sulfamethoxazole—two antibiotics that target folate (vitamin B9) synthesis—we predicted how *E*. *coli* could adapt to grow under folate-limited conditions. This work demonstrates how models can be used to study antibiotic resistance of drugs that target amino acid or cofactor synthesis.

## Introduction

Genome-scale metabolic models (M-model) are established approach for studying an organism’s metabolic capabilities. M-models have shown significant success in predicting the metabolic capabilities of a cell by integrating all the experimentally determined enzymatic reactions taking place in an organism [1–4]. These predictions are based on the stoichiometric constraints of the organism’s metabolic network and its metabolic interactions with the environment. Additionally, M-models often rely on an empirically derived biomass objective function which relates the growth rate of the simulation to the biosynthesis of all major biosynthetic building blocks needed to synthesize RNA, protein, and other macromolecules [5].

The use of this biomass objective function, however, implies that the abundance of all major components in a cell does not change based on growth rate or condition. In actuality, the macromolecular composition of a cell is highly dependent on its specific growth environment. This variability is due to the fact that the macromolecular composition of a cell is a function of the specific collection of proteins used to sustain growth in a particular environment. A key component of synthesizing a functional proteome—along with translating the amino acid chain and folding the peptides into their proper 3D structure—involves equipping enzymes with the necessary prosthetic groups and coenzymes. These accessory enzyme cofactors often drive the chemical conversions at the heart of an enzyme’s activity, making their presence essential for detectable catalytic activity [6,7]. The functions of some cofactors, such as flavins and iron-sulfur clusters, are so essential for core metabolism that their activity can be traced back to the beginning of life [8]. Thus, ensuring that all coenzymes and prosthetic groups are available to enzymes is essential for any robustly growing organism. The scarcity of one or more of the essential micronutrients can have a profound impact on the metabolic state of an organism, such as the disruption in energy metabolism and lactate secretion that is seen in *E*. *coli* growing in iron-limited stress conditions [9].

Despite their importance in sustaining metabolism, cofactor biosynthesis is not modeled mechanistically in M-models. This is due to the fact that cofactors are either enzyme prosthetic groups and thus have no modeled metabolic function (pyridoxine, biotin, etc.) or can be recycled (NAD, folates, etc.), meaning there is no metabolic process driving their biosynthesis. Thus, cofactors have often been incorporated into the biomass objective function to force the essential biosynthetic activity of enzymes forming these cofactors [5]. Biomass objective functions have been studied for M-models of various bacterial and archaea species providing insight into the essentiality of individual cofactors in prokaryotes [10]. However, even when included in the biomass objective function, a negligible amount of each cofactor is required to be synthesized for growth, causing cofactor synthesis to have little impact on metabolism overall. Furthermore, the specific requirement of the cofactors in the M-model is condition independent. Modeling efforts have been made to assess how the biomass function composition (lipid and amino acid composition) affects metabolic fluxes [11], but a mechanistic model has not been employed to relate cofactor demand to condition-dependent metabolism.

To that end, various methods have extended M-models to explicitly include the synthesis and use of the gene expression machinery, including coenzymes and prosthetic groups [12]. These modeling methods include resource balance analysis (RBA) [13,14], whole-cell modeling [15], flux-balance models that incorporate expression, thermodynamics, and resource allocation constraints (ETFL) [16], and other resource allocation models [17]. One such method to model resource allocation is termed genome-scale models of Metabolism and Expression (ME-models) [18–20], and they are capable of explicitly computing over 80% of the proteome by mass in enterobacteria. ME-models can provide a wide range of new biological insight including direct computations of proteome allocation [21], the effect of temperature on proteostasis [22], and the effect of membrane and volume constraints on metabolism [19]. Furthermore, their ability to compute the optimal proteome abundances for a given condition make them ideal for mechanistically integrating transcriptomics and proteomics data. Here we employ the *E*. *coli* ME-model [23] to examine the relationship between growth condition and cellular biomass composition. This work presents the first effort to apply a resource allocation model to comprehensively study the role that essential cofactors and other essential biomass components play in defining the metabolic capabilities of *E*. *coli*.

## Results

### Modifications to the ME-model

**Fig 1:**
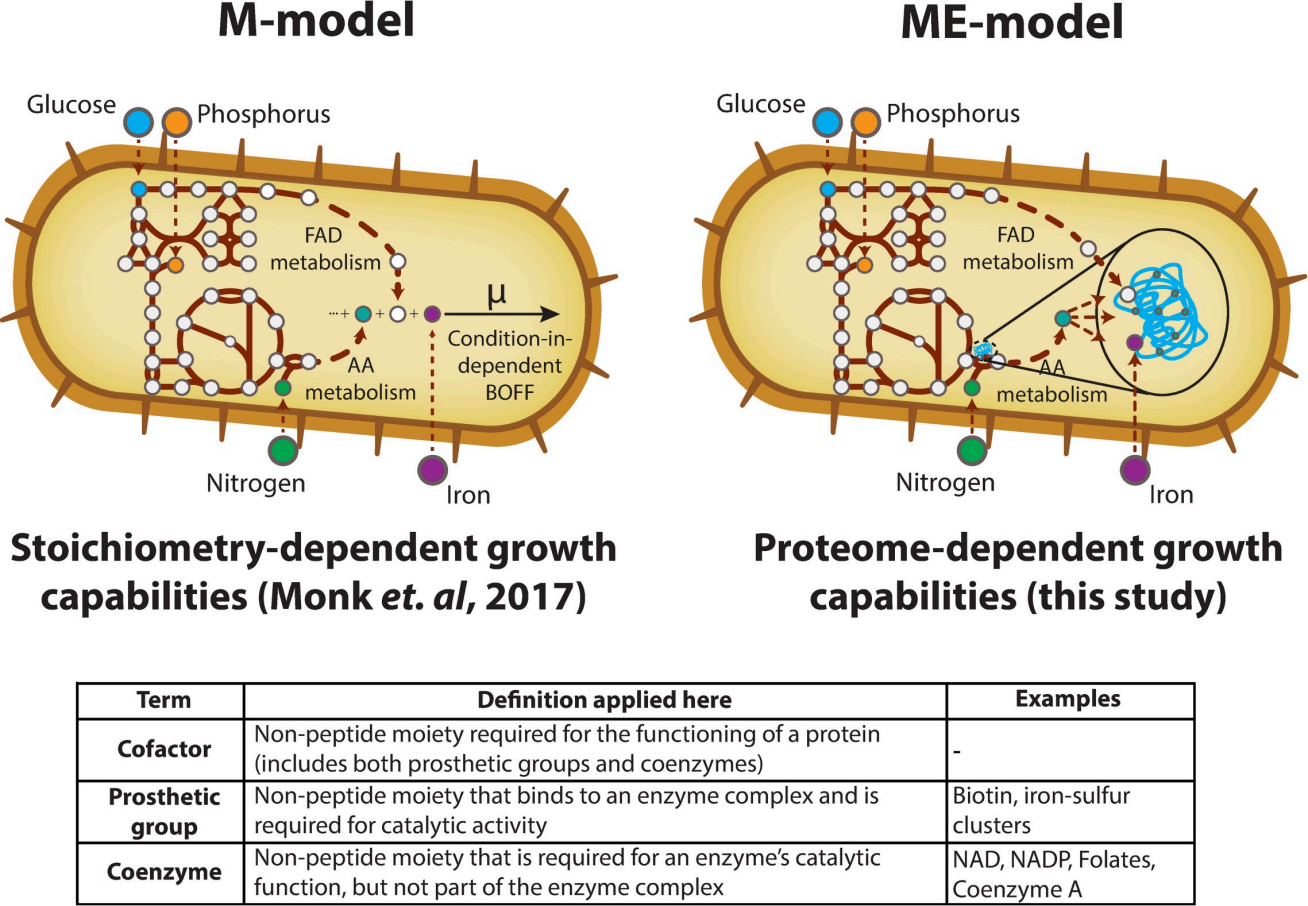
Difference in M- and ME-model scope. M-models offer a means to comprehensively probe the capabilities of enzymatic conversions possible within an organism. This modeling method is based on the stoichiometry of reactions in the organism’s metabolic network and can be used to predict possible growth supporting nutrient environments (demonstrated in Monk *et al*.) [25,26]. By mechanistically accounting for enzyme synthesis and activity, ME-models add additional information about the proteome sustaining the growth state. Thus, ME-models offer the ability to study how proteome allocation and cofactor use affects condition-dependent growth. Due to the inconsistency in how the terms **cofactor**, **prosthetic group**, and **coenzyme** are used in the scientific literature, the definitions applied in this study are listed in the table.

*E*. *coli* K-12 MG1655, along with many other microbes, are capable of *de novo* synthesizing all of the essential cofactors needed for growth listed in **Table 1**. Thus, the pathways that require these cofactors are included in both the *E*. *coli* K-12 MG1655 M-model (*i*JO1366 [24]) and ME-model (*i*JL1678b [23]). Unlike M-models, however, the activity of the prosthetic groups in **Table 1** is also explicitly modeled by the ME-model, as *i*JL1678b mechanistically describes all the processes required to produce a functioning proteome (**Fig 1**). Thus, for a particular enzymatic reaction to carry flux in the model, not only must the amino acids be synthesized in the proper proportions, but enzyme prosthetic groups must also be available. The condition-specific synthesis demand of both prosthetic groups and amino acids can therefore be assessed through ME-model computation.

**Table 1:** Summary of the vitamins synthesized by *E*. *coli* K-12 MG1655.

Cofactor Name (BIGG ID)	General Function	Cellular Role	Essentiality from Xavier *et al*. [10]	Vitamin
Thiamin (thm)	Energy metabolism	Prosthetic group	Universal	B1
Riboflavin (ribflv)	Redox metabolism	Prosthetic group/redox coenzyme	Universal (FMN, FAD)	B2
Niacin (nac)	NAD(P) precursor, electron carrier	Coenzyme	Universal (NAD, NADP)	B3
Pantothenoic Acid (pnto__R)	CoA precursor, fatty acid biosynthesis	Coenzyme	Universal (CoA)	B5
Pyridoxine (pydxn)	Versatile coenzyme that participates in transamination, decarboxylation, etc.	Prosthetic group	Universal	B6
Biotin (btn)	Required for carboxylase activity	Prosthetic group	Conditional	B7
Folate (thf)	Carrier of single carbon moieties	Coenzyme	Universal	B9
Cobalamin (cbl1)	Certain isomerases and methyltransferases, not essential in *E*. *coli* K-12 MG1655	Prosthetic group	Conditional	B12
Menaquinone 8 (mqn8)	Electron carrier	Coenzyme	Conditional (quinones)	K2
Ubiquinone 8 (q8)	Electron carrier	Coenzyme	Conditional (quinones)	-
2-Demethylmenaquinone 8 (2dmmq8)	Electron carrier	Coenzyme	Conditional (quinones)	-

Unlike prosthetic groups, coenzymes such as NAD and folates act as carriers that donate and accept energy (e.g., electrons carried by NAD(H)) or chemical moieties (e.g., single carbon groups carried by folates). These coenzymes are therefore regenerated throughout the network, meaning the synthesis of a coenzyme is not directly coupled to its activity. As is the case in M-models, coenzyme synthesis is not required for growth in the default ME-model and therefore is included as part of a “biomass constituent demand” reaction [23], analogous to the biomass objective function in M-models. *i*JL1678b was thus modified to account for the activity of these coenzymes and to couple coenzyme synthesis to its metabolic function (see **Materials and Methods**). Coenzymes were included in the analysis if they met 2 criteria: 1) they function in the cell exclusively as coenzymes within the *E*. *coli* metabolic network (i.e., cannot act as a metabolic precursor for biosynthesis) 2) they (or a very close derivative) are included in the *E*. *coli* wild-type biomass objective function. The activity of each coenzyme that met these criteria was determined based on the flux through the reaction that synthesizes the coenzyme (**Table A in S1 Text,** see **Materials and Methods**). Using this modified ME-model, growth simulations could effectively *de novo* predict the composition of *E*. *coli* K-12 MG1655’s appropriate biomass objective function in a condition dependent manner.

### Validating ME-model predictions of biomass composition

**Fig 2:**
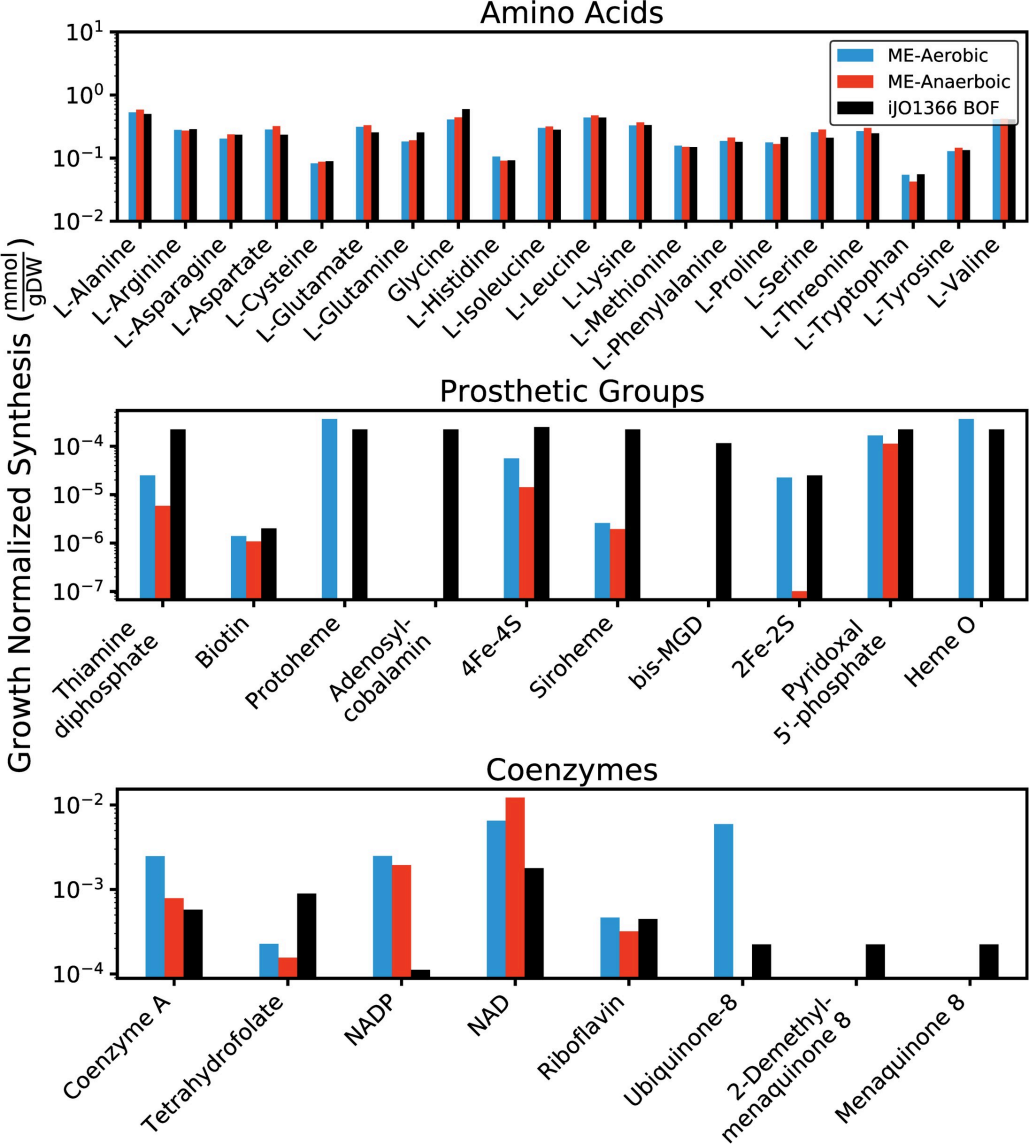
Comparison of growth-normalized ME- and M-model computed amino acid and cofactor synthesize rates. The ME-model biomass synthesis demands are a function of the predicted intracellular fluxes provided by the simulation, whereas the M-model values are provided by the biomass objective function. ME-model predictions are shown for aerobic and anaerobic *in silico* conditions.

With the model extensions outlined above, the ME-model was simulated under aerobic and anaerobic glucose M9 minimal media *in silico* conditions. The computed synthesis fluxes of amino acids, prosthetic groups, and coenzymes were then growth normalized to enable a comparison with the *i*JO1366 biomass objective function (**Fig 2**). The computed amino acid synthesis fluxes quantitatively agreed with the empirically derived numbers contained in the *i*JO1366 wild-type biomass objective function (BOF) and showed slight differences between aerobic and anaerobic simulations. This agreement suggests that amino acid composition of the proteome under multiple growth conditions is generally well represented by the BOF, though subtle changes in the amino acid composition could be observed depending on the growth condition.

Unlike amino acids, many of the cofactor requirements in the *i*JO1366 BOF are not derived from empirical data. In many cases, they are simply included with small coefficient values to ensure that the essential cofactor biosynthetic pathways are active in M-model computations [5,10]. Therefore, the *quantitative* comparison of the ME-model predicted cofactor usage to the M-model biomass objective function does not provide a high-confidence validation. This comparison does, however, confirm that the ME-model predicts the synthesis of most cofactors within a reasonable range, which is suitable for this study.

The predictions of cofactor composition provided by the ME-model are dependent on both the activity of specific reactions in a computed solution as well as the kinetic parameters used to couple reaction flux to enzyme abundance [27]. Thus, a stark difference in cofactor demand is expected when comparing two different computed metabolic states, which is observed for aerobic and anaerobic states (**Fig 2**). Reactions that are less utilized in anaerobic conditions, such as oxidative phosphorylation reactions and pyruvate dehydrogenase, see a decrease in their accompanying cofactors, ubiquinone 8 (q8) and thiamine diphosphate (thmpp, vitamin B1), respectively.

There are a few discrepancies between the *i*JO1366 BOF and ME-model predicted biomass compositions. For example, bis-molybdopterin guanine dinucleotide (bmocogdp), adenosyl-cobalamin (adocbl, vitamin B12), 2-demethyl menaquinone 8 (2dmmq8), and menaquinone 8 (mqn8, vitamin K2) are included in the wild-type objective function but have a predicted ME-model synthesis of zero for aerobic and anaerobic conditions. This discrepancy is partly because the wild-type objective function is formulated based on the wild-type biomass content of the cell, not the minimal set of biomass metabolites required for growth (represented by the “core” biomass objective function). The ME-model solutions, however, will predict only the minimal cellular content required for growth. Bis-molybdopterin guanine dinucleotide and adenosyl-cobalamin cofactors are not required for growth on glucose M9 minimal media and are thus not synthesized by the ME-model. 2-demethyl menaquinone 8 and menaquinone 8 are ancient naphthoquinone electron carriers that have been mostly associated with anaerobic growth, as opposed to ubiquinone 8 which is mostly used for aerobic growth [28]. The ME-model predicts the sole use of ubiquinone 8 for aerobic conditions and incorrectly predicts the use of no quinones for anaerobic growth. Naphthoquinones and ubiquinones have notably different redox potentials and therefore resource allocation models that incorporate thermodynamics could have the potential to address this discrepancy more closely [16].

### Growth condition-dependent biomass composition

The modified *i*JL1678b model was used to simulate growth on 557 nitrogen, phosphorus, sulfur, and carbon sources under aerobic and anaerobic *in silico* conditions (**S1 Table**). For these simulations, glucose M9 minimal media was used as the base *in silico* condition. Then the base carbon, sulfur, phosphorus, or nitrogen source was supplemented with one of the 557 metabolites, and the model was optimized. To facilitate a comparison across a diverse set of *in silico* conditions, the computed biosynthesis demand of each cofactor and amino acid was normalized by the total protein biomass predicted for the condition (see **Materials and Methods**). The computed micronutrient demands from the 592 growth-supporting simulations effectively provided condition-dependent biomass compositions predicted *de novo* from the ME-model.

**Fig 3:**
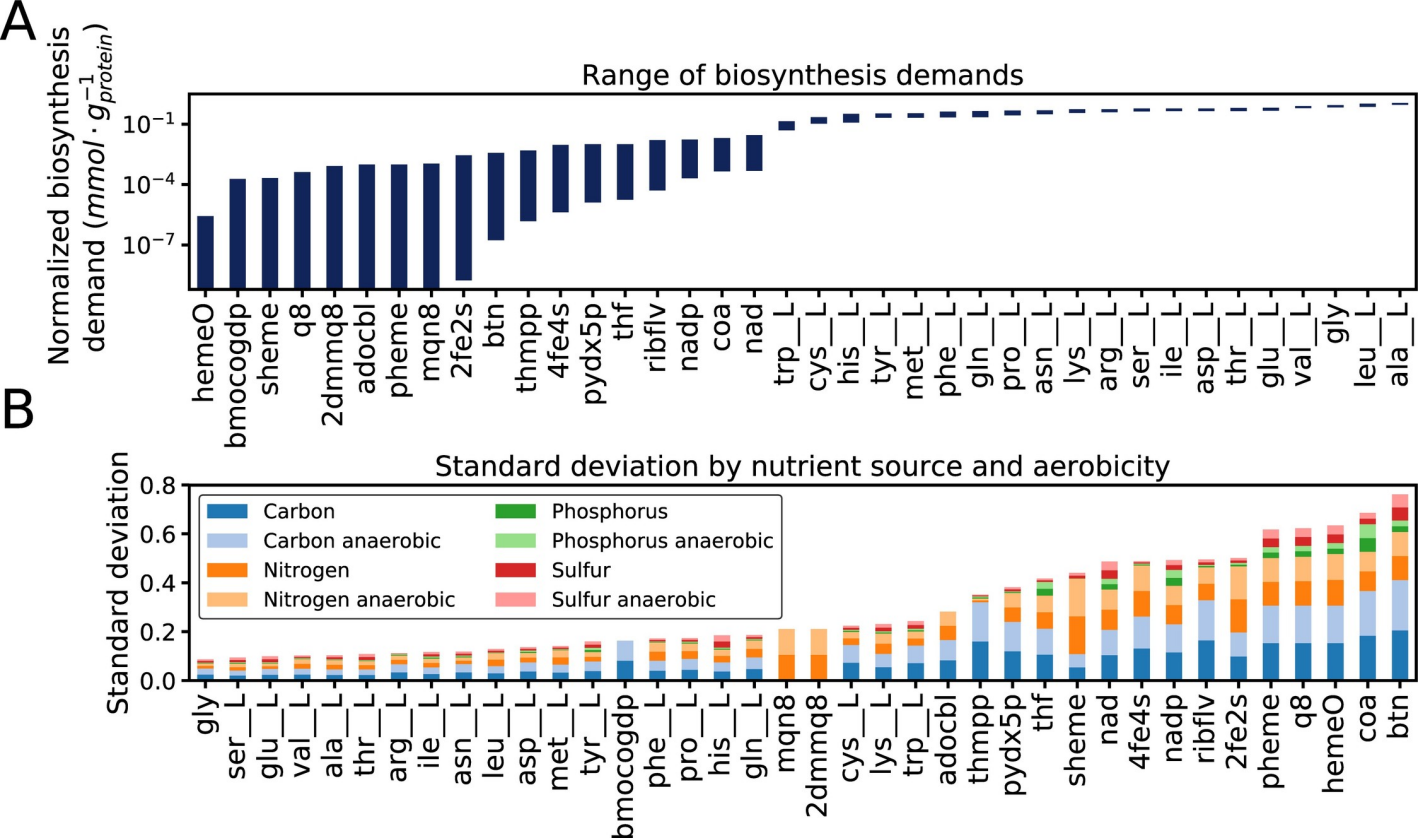
Variation in the synthesis demand (i.e., the amount of each micronutrient that must be synthesized to sustain growth) of enzyme cofactors and amino acids by growth condition. **A)** The maximum and minimum biosynthesis demand for each amino acid and cofactor across all growth conditions. **B)** Stacked bar chart showing the standard deviation in normalized synthesis demand of each nutrient source and aerobicity.

Variability was observed for many of the micronutrients depending on nutrient source and aerobicity. This variability was particularly notable for the cofactors, which were found to vary by several orders of magnitude (**Fig 3A,** using BiGG IDs [29] shown in **Table B in S1 Text**). The range of amino acid biosynthetic demands across conditions, however, were much narrower. The standard deviation of each micronutrient was further observed as a function of nutrient source and aerobicity (**Fig 3B**). These computations showed that the variation in cofactor use was largely driven by the carbon and nitrogen sources used for the simulations. Carbon and nitrogen sources also displayed greater variation in the computed growth rates (**Fig A in S1 Text,** refer to **Materials and Methods** for a discussion of model parameterization and growth rates). Little variation in micronutrient demand and growth rate was observed among phosphorus and sulfur sources. Furthermore, **Fig 3B** suggests that aerobic and anaerobic conditions show a similar amount of biomass composition variability.

Furthermore, a subset of the enzyme cofactors was computationally predicted to be required only in specific growth conditions. The use of some of these cofactors (e.g., ubiquinone-8, protoheme (pheme), and heme O (hemeO)) differed based on the aerobicity of the model simulations (**Fig B in S1 Text**). This behavior is expected for these cofactors, as they are primarily required for aerobic respiration functions. The demand of other cofactors such as bis-molybdopterin guanine dinucleotide (bmocogdp), adenosylcobalamin (adocbl), and siroheme (sheme) are specific to individual growth conditions. For example, bis-molybdopterin guanine dinucleotide is a molybdopterin-containing prosthetic group required for numerous membrane-bound oxidoreductases used for respiration on non-oxygen electron acceptors and for detoxifying oxidation products of biotin and methionine [30]. Among the oxidoreductases that require bis-molybdopterin guanine dinucleotide is formate dehydrogenase (FDH) [31] used by the *in silico* cell when the primary carbon source is a derivative of formate, glycine, or a purine. FDH is also required for the catabolism of urate [32,33]. Adenosyl-cobalamin is computationally required for growth only when the carbon or nitrogen source is ethanolamine, as adenosyl-cobalamin in an essential cofactor for ethanolamine ammonia-lyase, the first step of ethanolamine catabolism. Siroheme is computationally required in most growth conditions for the activity sulfite reductase. (SULR), an essential step in the reduction of sulfate to hydrogen sulfide for sulfur assimilation. Growth on other sulfur sources, such as cysteine and cysteine derivatives, is computationally predicted to alleviate the need for siroheme.

#### Characterizing the aerobic and anaerobic growth by predicted biomass composition

**Fig 4:**
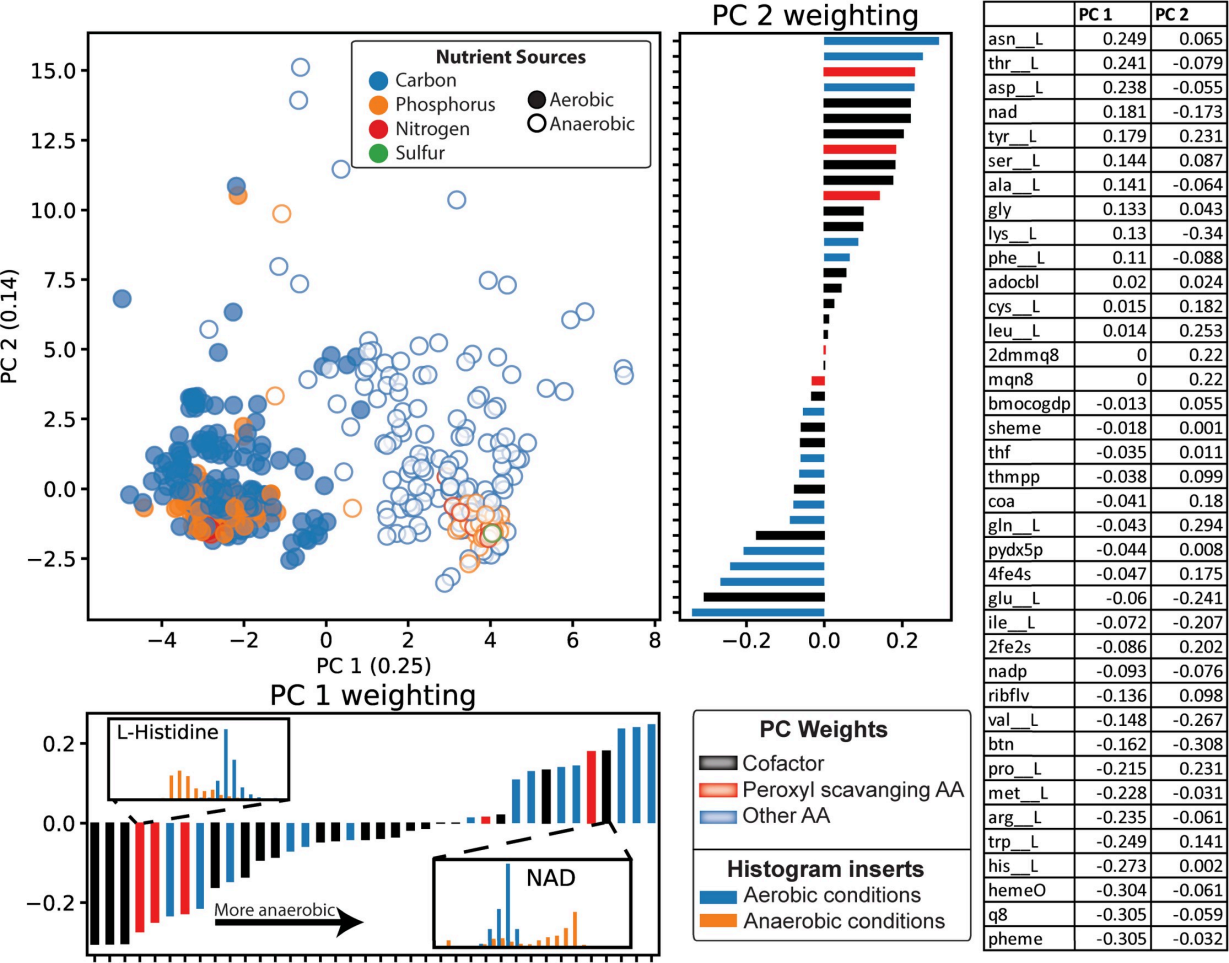
Differences in the synthesis demand of enzyme cofactors and amino acids by aerobicity. PCA analysis of all computed growth conditions reveals aerobic (filled points) and anaerobic (outlined points) growth conditions can be resolved by principal component 1. The protein biomass normalized micronutrient demand for L-histidine and NAD are shown next to their component 1 weighting. The two histograms demonstrate the clear separation in aerobicity-dependent demand of these micronutrients. A table of the principal component vector weightings is shown on the right.

The differences in biomass composition between aerobic and anaerobic growth conditions were also analyzed. PCA decomposition of the computed micronutrient demands showed that aerobic (filled points) and anaerobic (outlined points) simulations could be differentiated along principal component 1 (**Fig 4**). Principal component 1 thus describes the biomass constituents with demands that differ based on the aerobicity of simulation. Further investigation of the vector weightings of principle component 1 suggested a general decrease in cofactor use for anaerobic conditions relative to aerobic growth. The cofactors most negatively weighted in component 1 (favored in aerobic conditions) include ubiquinone-8 (q8), protoheme (pheme), and hemo O (hemeO) which are cofactors highly involved in the electron transport chain in aerobic conditions. Alternatively, one of the few cofactors that was positively weighted in component 1 (favored under anaerobic conditions) was NAD. This observation is expected given the increase in the rate of glycolysis (and thus NAD turnover) observed in fermentative anaerobic metabolism. The distinct increase in the use of NAD in anaerobic solutions is shown as a histogram in **Fig 4.**

Some amino acids appeared to be preferentially utilized in aerobic conditions. Three of the four most negatively weighted amino acids in component 1 are L-histidine, L-tryptophan, and L-methionine, suggesting the proteomes used in aerobic conditions are enriched in these amino acids. These three amino acids are peroxyl scavenging amino acids, and thus proteins enriched in these amino acids could have more redox reactivity [34]. In fact, it has been hypothesized that the diversification of amino acids was in part driven by the presence of oxygen and its oxidative properties [34]. It is therefore unsurprising that aerobic metabolic states would utilize proteins containing these three amino acids. Furthermore, L-histidine is effective at forming metal ion ligation sites for metalloproteins [35]. Metalloproteins, particularly those with iron metal centers [36], are needed to sustain the increase in redox reaction activity that occurs in aerobic conditions. Therefore, the increase in L-histidine residues-containing proteins could reflect the increase in metalloprotein use in aerobic growth.

Principal component 2, on the other hand, does not appear to be associated with the aerobicity of the simulation but it is well correlated with growth rate (⍴ = -0.50, p-value = 2.99 x 10^−40^). This suggests that this component could capture the components in the model that are still dependent on growth rate even following the normalization by computed protein biomass.

#### Clustering growth conditions by predicted biomass composition

**Fig 5:**
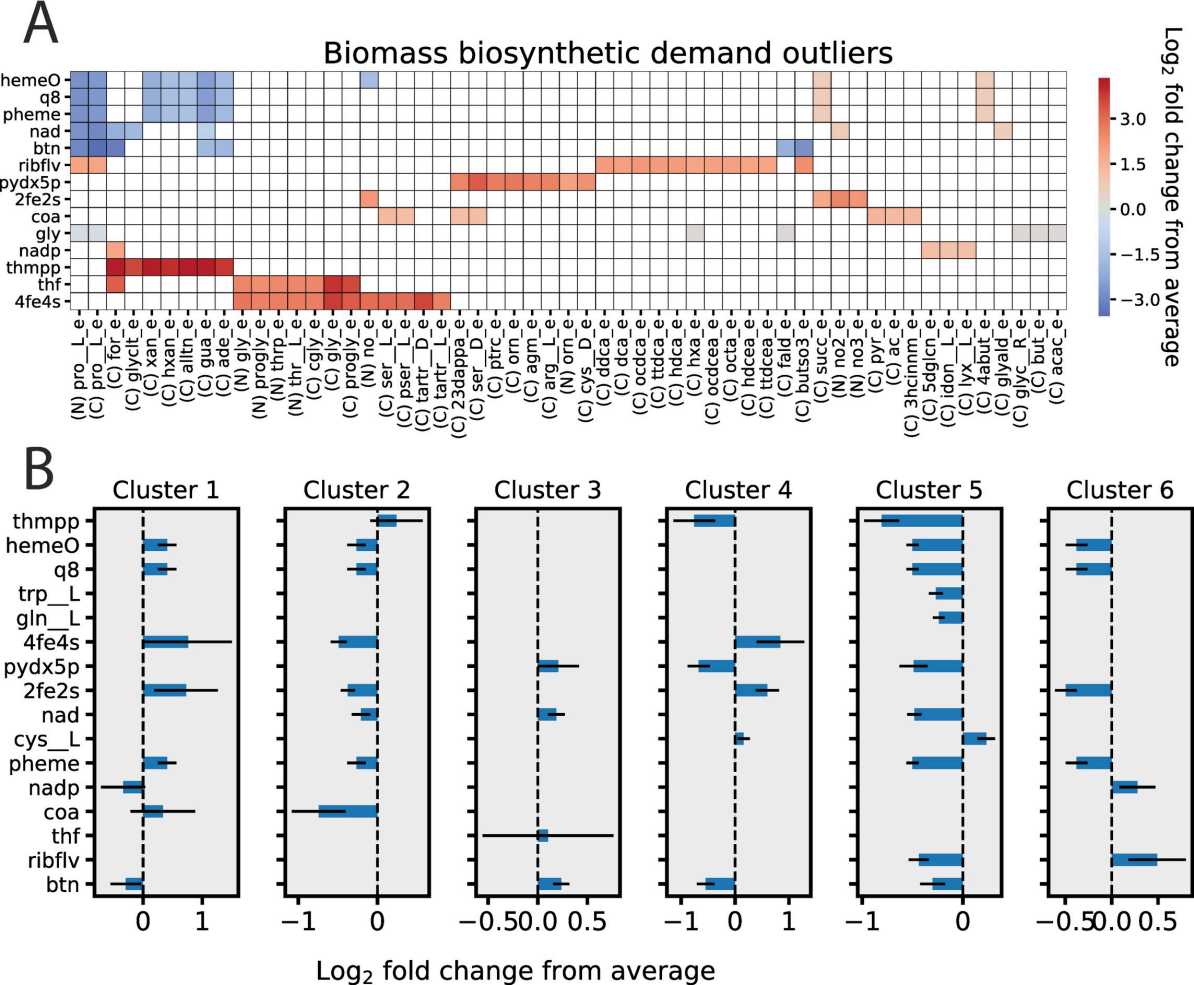
Characterization of aerobic condition-dependent biomass compositions. **A)**. Outlier analysis was performed to find growth conditions with z-scored biomass constituent synthesis demands with absolute values greater than 3. The colors on the heatmap denote the log_2_ fold change of the outlier compared to the average biosynthetic demand of all aerobic growth conditions. **B)** Hierarchical clustering using Ward’s linkage was performed to divide the *in silico* growth conditions into 6 clusters based on their predicted biomass demands. All 6 clusters contained multiple biomass demand values that were statistically different (p < 1x10^-5^ by Wilcoxon rank-sum test and log_2_ fold change >0.15) compared to the non-cluster demands. The significant log_2_ fold changes are shown comparing growth conditions in each cluster compared to the average for growth conditions not in the cluster. Error bars represent the standard deviation of the log_2_ fold change values.

Having characterized the major differences in computed biomass compositions during aerobic and anaerobic growth, we next focused on more deeply characterizing differences among the aerobic *in silico* growth conditions. It was shown in **Fig 3** that the nutrient source variation was similar for aerobic and anaerobic conditions, and thus only aerobic conditions were used to simplify analysis. First, univariate analysis was performed to identify growth conditions that resulted in uniquely high or low biomass demand needed for growth. The normalized biomass demands for the 330 aerobic growth supporting conditions were z-scored and absolute values greater than 3 were selected as outliers. The heatmap in **Fig 5A** shows these identified outliers as well as the accompanying log_2_ fold change from the mean.

Multiple growth conditions computationally required a much higher folate (thf, vitamin B9) biosynthesis demand than the remaining conditions. These conditions include glycine (gly) and prolylglycine (progly) as carbon or nitrogen sources, formate (for) and L-cysteine-L-glycine (cgly) as carbon sources, and L-threonine O-3-phosphate (thrp) and L-threonine (thr__L) as nitrogen sources. On average these growth conditions require 8-fold higher folate demand than the average of all remaining conditions. Wild-type *E*. *coli* cannot utilize formate or glycine as sole carbon sources, but *E*. *coli* growth using formate has been engineered by overexpressing formate-tetrahydrofolate ligase along with other components of the folate and serine cycles of *Methylobacterium extorquens AM1*. Further adaptive laboratory evolution of this strain showed that mutations in the rate limiting step of folate biosynthesis was necessary for robust growth, presumably to increase the rate of folate synthesis [37]. The model suggests that growth on glycine would require a notable increase in the activity of the folate-dependent glycine cleavage system, which could potentially be obtained through adaptive laboratory evolution.

Additionally, computed pyridoxal phosphate (pydx5p, vitamin B6) demand is significantly higher (6.6-fold higher than remaining nutrient sources) when 2,3-diaminopropionate (23dappa), D-serine (ser__D), putrescine (ptrc), agmatine (agm), L-arginine (arg__L), D-cysteine (cys__D) are carbon sources and when L-ornithine (orn) is a nitrogen or carbon source. Thus, growth on this set of nutrients uniquely requires high activity of enzymes with pyridoxal phosphate prosthetic groups. Four nutrient sources (L-arginine, putrescine, agmatine, and L-ornithine) are key precursors or intermediates in L-arginine or L-ornithine metabolism. Pyridoxal phosphate has an important role in amino acid metabolism as a common cofactor for transaminase reactions [38]. Therefore, it is logical that pyridoxal phosphate will be highly involved in the metabolism of L-arginine (a urea cycle component) and other derivatives that are highly involved in nitrogen metabolism, particularly in humans.

The remaining three growth conditions (D-serine, D-cysteine, and 2,3-diaminopropionate) are outliers due to activity of unique pyridoxal phosphate-containing enzymes such as 2,3-diaminopropionate ammonia lyase (DAPAL) [39], D-serine deaminase (SERD_D) [40], and D-cysteine desulfhydrase (CYSDDS) [41] which are required to metabolize 2,3-diaminopropionate, D-serine, and D-cysteine, respectively. Interestingly, only the D-stereoisomers of serine and cysteine are outliers for pyridoxal phosphate synthesis. For the case of serine, serine racemase (SERR) is not present in *E*. *coli* to convert D- to L- serine directly, causing these two stereoisomers to be metabolized differently. L-serine, in fact, is an outlier for a different set of biomass components than D-serine including coenzyme A (coa, vitamin B5) and 4Fe-4S (4fe4s) iron sulfur clusters. This is because L-serine deaminase (SERD_L) requires a 4Fe-4S iron sulfur cluster cofactor instead of pyridoxal phosphate. Alternatively, D-cysteine has a high computational pyridoxal phosphate requirement due to D-cysteine desulfhydrase (CYSDDS) activity. L-cysteine desulfhydrase (CYSDS) also requires pyridoxal phosphate for activity [42] but is incorrectly annotated in the ME-model. Correcting this annotation error will be a priority in future model development.

Also of note are 10 fatty acid carbon sources (e.g., hexanoate (hxa), tetradecenoate (ttdcea), etc.) which are distinguished by the high demand for riboflavin (ribflv, vitamin B2) needed for growth on these substrates. Wild-type *E*. *coli* K-12 MG1655 is capable of growing only on long chain fatty acids, though *E*. *coli* mutants exist that are capable of growing on short and medium chain fatty acid as well [43]. This is largely dependent on the strain’s basal expression of β-oxidation enzymes, which often require FAD, a riboflavin containing cofactor.

The outlier conditions in **Fig 5A** were removed and the remaining aerobically computed biomass compositions were normalized by the maximum cofactor or amino acid demand (see **Materials and methods**). Using hierarchical clustering, these 274 growth supporting conditions were partitioned into 6 groups based on the similarity of their computed biomass composition (**Table 2**). The differences in the biomass composition of the growth conditions could be examined in greater detail if the number of clusters was further increased, but 6 clusters was found to optimize the gap statistic for the computed solutions [44]. The 6 clusters range in size from 12 to 161 growth conditions and represent groups of conditions that exhibit distinct *in silico* biomass compositions. The biomass compositions for the conditions within each of these clusters could therefore be considered different enough to necessitate a unique biomass objective function (**S2 Table**). The clusters were characterized by performing a Wilcoxon rank-sum test on the micronutrient demands of conditions in the cluster compared to all conditions outside of the cluster. The biomass demands that were significantly different (p < 1x10^-5^ and absolute log_2_ fold change >0.15 relative to the non-cluster average) for each cluster are summarized in **Fig 5B**.

**Table 2:** Clustering characterization.

Cluster	Cluster summary	Number of conditions in cluster
1	TCA cycle intermediates and derivatives	29
2	Nucleotides and derivatives as carbon sources	41
3	Glucose and other nutrients with minimal impact on biomass composition (most phosphorus and sulfur sources)	161
4	D-alanine and misc. non-glucose sugars	13
5	Deoxynucleotides as carbon sources	12
6	Nucleotides and derivatives as nitrogen sources	18

The largest cluster was cluster 3 which contained growth substrates computationally metabolized with proteome compositions similar to the default growth environment (i.e., glucose M9 *in silico* media). This cluster represented the growth substrates that are predicted to have a minimal impact on the computed biomass composition relative to the default growth state. Cluster 3 included the vast majority of the phosphorus and sulfur sources, consistent with the observation in **Fig 3B** that these sources cause little variability in the biomass composition. These are the conditions in which a biomass objective function obtained from growth on glucose M9 minimal media would be most applicable.

Interestingly, nucleotide nutrient sources partition into three different clusters (Clusters 2, 5, and 6 **Table 2**). Cluster 2 contained 41 growth conditions, most of which were conditions where the primary carbon source is a nucleotide or nucleotide derivative. Cluster 5, however, contained 12 growth conditions where the primary carbon source is the deoxynucleotide version of many of the nucleotides in Cluster 2. Despite the minor difference in chemical structure for the metabolites in the two clusters, they differed greatly in the biomass constituents used for their metabolism (**Fig 5B**). For example, the deoxynucleotide required ~50–25% less thiamine diphosphate (thmpp, vitamin B1), riboflavin (ribflv, vitamin B2), and biotin (btn, vitamin B7) due to lessened computed pyruvate hydrogenase and lipid metabolism activity when metabolizing deoxynucleotides. Metabolizing the deoxynucleotides in Cluster 5 additionally required a proteome containing 18% more L-cysteine content than the proteome of metabolites outside of Cluster 5. This increase in L-cysteine content implies that metabolizing the metabolites in Cluster 5 requires the expression of more proteins with active sites involved in proton shuttling or covalent catalysis through nucleophilic interactions [45,46].

### Multi-scale analysis of micronutrient limitation in *E*. *coli* metabolism

As demonstrated above, resource allocation models such as ME-models offer the ability to comprehensively study how the metabolic needs of an organism can directly influence its use of essential biomass constituents. Alternatively, ME-models can be applied to understand the opposite relationship: the relationship between biomass constituent availability and the metabolic state of the organism. These biomass components, particularly cofactors, are involved in many important cellular functions, thus their activity can have a profound impact on the cellular phenotype [47,48].

Despite the importance of these biomass components for a cell’s metabolic sustainability, many strains of *E*. *coli* have lost the ability to synthesize some of these cofactors and amino acids throughout their evolutionary history [49]. The evolution of auxotrophy is commonly observed in clinical strains of *E*. *coli*, and thus understanding the metabolic consequences of auxotrophy can lead to a better understanding of the interactions between pathogenic microbes and their host environment [26,50]. The ME-model was applied to study *E*. *coli* auxotrophs under *in silico* conditions where availability of the essential metabolite is limited. The predicted cellular response was studied on three levels of resolutions: a phenotypic level, a subsystem level, and by observing changes in the activity of individual reactions.

#### Characterizing growth under micronutrient limitation

There are multiple physiological scenarios where bacteria could face growth in excess or limitation of one of the essential biomass constituents studied here. Conditions of nutrient excess (**Fig C in S1 Text**) can include growth in the human gut, while nutrient limited conditions can include growth under antibiotic stress (e.g., growth in the presence of antifolates) or auxotrophic growth in conditions of nutrient limitation. Given *E*. *coli*’s notable capabilities to evolve and adapt to growth under stress, we apply the ME-model to predict how *E*. *coli* could adapt to nutrient limitation by reallocating its existing proteome.

**Fig 6:**
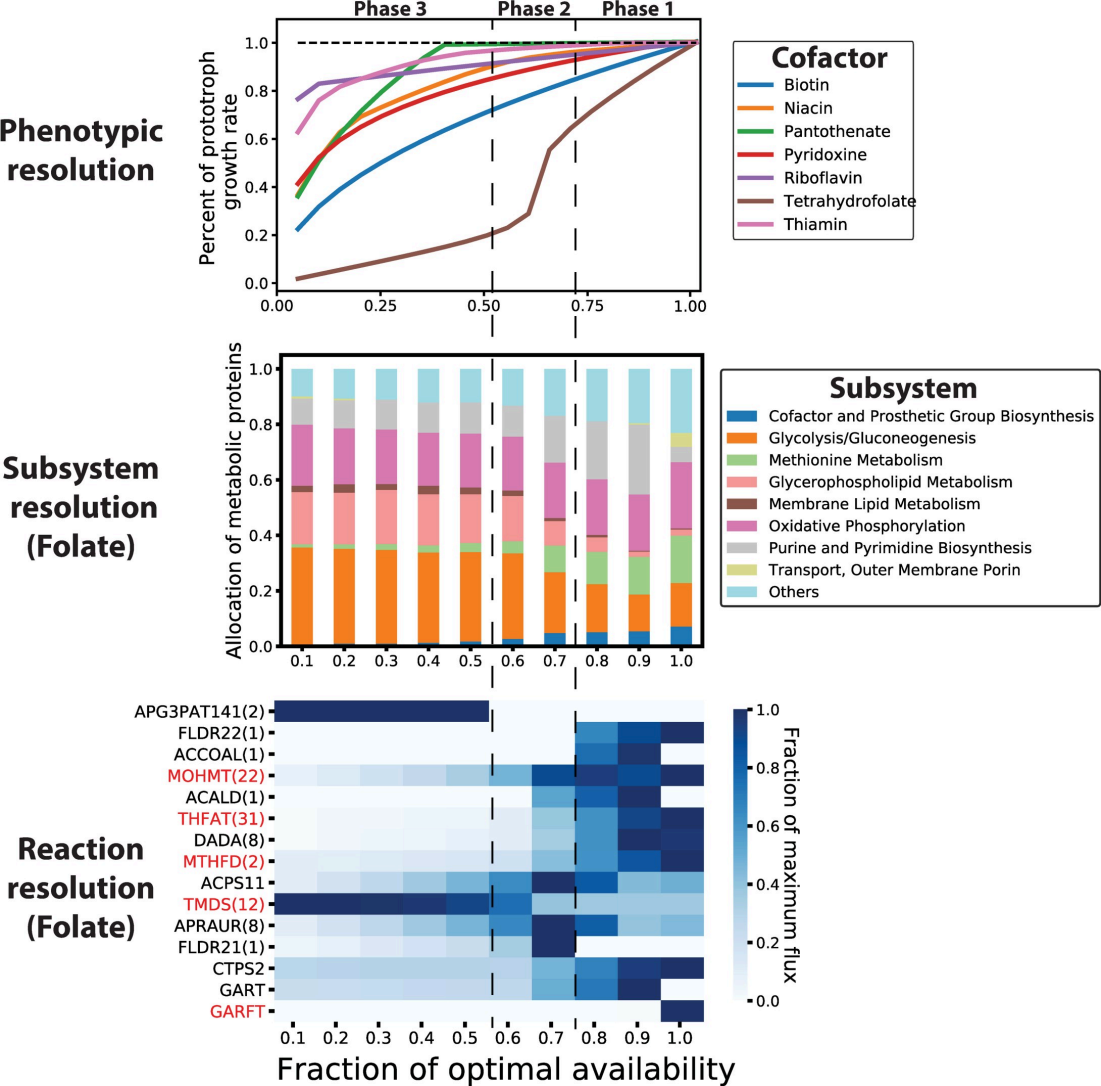
Computed growth rate in auxotrophic models of *i*JL1678b when the availability of the essential cofactor in the legend is limited. For each of the 7 metabolites shown in the legend, reactions were imposed into the model creating an auxotrophy for that metabolite (**Table C in S1 Text**). **Top panel**: The growth rate is plotted as a function of the availability of the metabolite indicated in the legend. The percent change in the growth rate compared to the wild-type (prototroph) model is shown. **Middle panel:** Model-predicted metabolic changes in response to tetrahydrofolate limitation. The mass fraction of protein allocated to each metabolic subsystem during tetrahydrofolate limitation is shown (columns) **Bottom pane**l: Heatmap showing fraction of maximum growth rate-normalized reaction fluxes. The 15 reactions with the highest standard deviation are shown and are highlighted in red if the reaction relies on folate activity. If reaction fluxes were perfectly correlated throughout the tetrahydrofolate limitation simulations, then these reactions were grouped together. The number in parentheses shows the number of other reactions represented by the row. The right column depicts a simulation with the highest folate availability and the left column depicts a simulation with the lowest folate availability.

Reactions were knocked out that rendered the ME-model auxotrophic for 11 essential biomass components (**Table C in S1 Text**). To gauge how nutrient limitation of these biomass components impacts growth, *in silico* growth rates were observed as a function of the uptake rate of each of the 11 essential amino acids or cofactors. Optimal growth rates were computed as a function of essential metabolite availability. For these simulations, the uptake rate of each essential metabolite was determined at the maximum growth rate. The auxotrophic model was then optimized with the metabolite uptake rate constrained to values ranging from 100% to 5% the uptake rate at maximum growth. The simulations predicted that, while amino acid limitation elicited a consistent response in the computed growth rate, there was notable variability in the response of the *in silico* cells depending on the essential cofactor (**Figs D and E in S1 Text**). Most notably, tetrahydrofolate auxotrophs were predicted to be particularly growth sensitive to drops in tetrahydrofolate availability below the optimal amount (**Fig 6**). This drop in growth occurred in three phases, one in which growth drops gradually to 55.5% of the maximum as folate availability decreased to 65.0% of the optimal. The second phase consists of the sharp decrease in growth from 55.5% to 28.9% of the maximum growth as folate availability decreased from 65.0% to 60.0%. The third phase displayed a gradual decrease in the computed growth rate to 0.

The change in protein allocation underlying folate limitation and the resulting drop in growth rate was also assessed. This analysis was performed using ME-model predictions of protein abundance and the metabolic subsystem annotations provided by *i*JO1366. In the first phase of folate limitation, the protein allocated to various subsystems was highly variable, with the amount of protein allocated to purine and pyrimidine metabolism notably increasing. During the drop in growth in phase 2, protein allocation to methionine metabolism, cofactor biosynthesis, and purine and pyrimidine biosynthesis dropped and was reallocated to glycolysis and glycerophospholipid metabolism. The third phase was marked by relatively consistent protein allocation with a minor increase in methionine metabolism and cofactor biosynthesis. The ME-model thus shows a disruption (and thus a compensation by increasing protein allocation) in purine and methionine metabolism, which is known to occur upon treatment with antifolate antibiotics [51,52].

Using the ME-model, metabolic changes can be observed at a higher resolution by characterizing the individual reactions driving the global changes described above (**Fig 6**). For example, when folate is optimally available, the phosphoribosylglycinamide formyltransferase (GARFT, *purN*) reaction is active which uses formyl-tetrahydrofolate (10fthf) to produce N2-Formyl-N1-(5-phospho-D-ribosyl)glycinamide (fgam), an important intermediate in purine biosynthesis. Folate limitation results in a transition to using GAR transformylase-T (GAR, *purT*), an ATP driven reaction that can produce fgam from free formate. The shift from using GARFT to GART likely contributes to the increase in protein allocation needed for purine metabolism. Folate inhibitors have in fact been shown to significantly impact the activity of this metabolic node, leading to rapid accumulation of N1-(5-Phospho-D-ribosyl)glycinamide (gar) and depletion of fgam [53]. However, *purN* activity was shown to be essential for *E*. *coli* survival during antifolate treatment, potentially to recycle tetrahydrofolate from the cellular reserves of formyl-tetrahydrofolate. Additionally, the large drop in growth observed in phase 2 of **Fig 6** coincides with a decrease in 3-methyl-2-oxobutanoate hydroxymethyltransferase (MOHMT), an essential step in coenzyme A biosynthesis.

Alternatively, a similar analysis was performed for niacin limited growth states (**Figs F and G in S1 Text**). In a niacin limited environment, the cellular pools of reduced and oxidized NADP and NAD would be highly depleted. Therefore, an optimally growing cell in this state would likely have to redirect flux into pathways that maximize growth while requiring less activity of these two cofactors. *i*JL1678b predicts that the optimal approach to optimize NAD and NADP use is: 1) to upregulate the Entner-Doudoroff pathway (bypassing lower glycolysis) 2) to increase activity of the glyoxylate shunt to donate electrons to the quinate pool via malate and 3) to donate electrons to the quinate pool via formate (pyruvate formate lyase (PFL) and formate dehydrogenase (FDH4pp)) and lactate (D-lactate dehydrogenase (LDH_D2)). It is unclear the metabolic route to donate electrons from formate is feasible since PFL is typically only expressed in anaerobic conditions in *E*. *coli* K-12 MG1655 [54]. Given the rise in antimicrobial resistance to some antifolates antibiotics, understanding metabolic strategies for tolerating nutrient stress could provide insight how to combat this resistance through possible combinatorial therapies or to potentiate the antibiotic’s activity.

## Discussion

The presented work provides the first computational study applying resource allocation models to highlight the systems-level interplay between *E*. *coli*’s condition-dependent metabolic state and biomass composition. The ME-model of *E*. *coli* K-12 MG1655 is one such resource allocation model suited to examine this relationship, given that it inherently provides predictions of the functional proteome required to sustain a particular metabolic phenotype, including cofactor usage. Using the *i*JL1678b model, simulations were performed on all growth supporting nutrients in the model, demonstrating notable variability in the computed biomass composition depending on the specific growth environment. To determine the metabolic consequences of acquired auxotrophy (or nutrient limitation through antibiotic treatment), the relationship between metabolism and cofactor or amino acid availability was examined. These results provide insight into the metabolic deficiencies that could accompany a drop in the availability of essential nutrients.

After validating the ME-model predicted biomass objective functions (**Fig 2**), simulations were performed for growth on 557 nutrient conditions aerobically and anaerobically, thus providing 592 *de novo* predictions of condition-dependent biomass objective functions. Analysis of these biomass compositions suggested that unique biomass functions could be appropriate for anaerobic and aerobic simulations. For example, an aerobic objective function could include an increase in the abundance of peroxyl scavenging amino acids such as L-histidine, L-tryptophan, and L-methionine along with an increase in most enzyme cofactors, with NAD being the exception (**Fig 4**).

ME-models can predict new methods for improving the efficacy of antibiotics either by manipulating the microenvironment of the bacteria or suggesting combinatorial drug therapies. Given that B vitamins, such as folate (vitamin B9), provide good targets for antibiotic treatments, the model predictions of growth condition-dependent cofactor demand could be useful for designing cellular microenvironments to increase or decrease the susceptibility of *E*. *coli* to some antimicrobials. For example, trimethoprim and sulfamethoxazole are antibiotics that inhibit the synthesis of tetrahydrofolate via inhibition of dihydrofolate reductase and dihydropteroate synthetase, respectively. Trimethoprim is commonly used to treat urinary tract infections, but ~30% of infections in the US have been shown to be resistant to this antibiotic [55]. The analysis in **Fig 5** suggests that treating *E*. *coli* with antifolates in a glycine rich environment could potentiate the effect of the antibiotics by increasing the cellular demand of folates. Likewise, the predicted metabolic response to folate limitation could suggest combinatorial therapies for antimicrobials that target the production of these cofactors. The results in **Fig 6** predict that disrupting the activity of the phosphoribosylglycinamide metabolic node could improve the efficacy when treating *E*. *coli* infections with antifolates.

The presented work contains several notable limitations. First, the computed synthetic demand for each biomass component was normalized by the computed protein biomass. This normalization works well for amino acids and prosthetic groups, whose synthetic demands are a function of protein abundance. However, coenzyme (e.g., NAD, folate, etc.) usage is not necessarily a function of protein abundance. Future work should be done to normalize and analyze these classes of biomass components separately to gain a more nuanced view of their condition-dependent activity. Second, a pseudo-kinetic term with a constant value was applied to couple the activity of coenzymes to their biosynthetic demand. This approach was sufficient for the current study as we sought to characterize only the differences in cofactor use across conditions. Future studies to characterize coenzyme demand at a quantitative level should estimate this term from experimental data and perform a sensitivity analysis around the estimated value. Last, this work provides a new look into the inherent coupling between cell synthesized cofactors and metabolism. However, a separate integral part of a functional proteome is the metal ion cofactors that form the enzymatic center of many enzymes. Due to the interchangeability of some ion cofactors and intricate mechanisms underlying enzyme mismetallation, fully examining metal ion cofactors was out of the scope of this study [56]. Future work is warranted to study the metalloproteome and how metal ion availability shapes metabolism.

Lastly, the predictions from this computational study are well suited for future experimental validation. First, many of the cofactor and amino acid auxotrophs are the product of only single gene knockouts in *E*. *coli* K-12 MG1655, meaning these strains either already exist in single knockout libraries [57] or can be easily synthesized. Adaptive laboratory evolution of these auxotrophs in low concentrations of their essential nutrients could provide valuable insight into mechanisms of *E*. *coli* adaptation to re-invest its protein toward pathways that maximize growth, while minimizing cofactor use. Second, this work provides predictions of ways to manipulate the growth environment of *E*. *coli* to potentiate the effect of antibiotics. Future testing of these predictions *in vivo* would further underscore the utility of this modeling method.

## Materials and methods

### Software

All constraint-based modeling analyses were performed using Python 3.6 and the COBRApy software [58]. ME-model operations were performed using the COBRAme framework [23]. Since ME-models are ill-scaled [18], qMINOS [59,60], which supports quad (128-bit) precision, was used for ME-model simulations. M-model simulations were performed using the *i*JO1366 model of *E*. *coli* K-12 MG1655 metabolism [24], since *i*JL1678b-ME was reconstructed using this M-model of *E*. *coli* as a scaffold. All M-model optimizations were performed using the Gurobi (Gurobi Optimization, Inc., Houston, TX) linear programming (LP) solver.

### ME-model modifications for modeling cofactor activity

The *i*JL1678b ME-model of *E*. *coli* K-12 MG1655 was used for all simulations in this study. The activity of enzyme prosthetic groups is inherent in the ME-model formulation [18], which uses coupling constraints to connect the synthesis of individual enzymes (including their accessory groups) to the reactions they catalyze. Coenzymes (NAD, folates, etc.) have some of the same properties as enzymes in that they are recycled within the network in both M- and ME- models. These models therefore ensure that the coenzymes are balanced, but they do not require the biosynthesis of these coenzymes. As a result, models have incorporated these coenzymes in the biomass objective function to force their biosynthesis in a way that is independent of their use in the model.

The *i*JL1678b ME-model was thus modified to couple the biosynthesis of coenzymes to their activity, similar to other enzymes in the model. This is accomplished using a pseudo-kinetic term to relate the concentration of the coenzyme pool to its activity in the metabolic network, which we will simply call k_activity_. This term represents a very rough estimation of the first-order kinetics of the reactions involving the coenzyme in the network. This term was chosen as 1x10^4^ hr ^-1^ and applied to each reaction where the uncharged version of the coenzyme acts as a reactant:

$$\left(1+\frac{\mathrm{mu}}{k_{\mathrm{activity}}}\right)\mathrm{uncharged}\_\mathrm{coenzyme}+\mathrm{met}\_1\to \mathrm{charged}\_\mathrm{coenzyme}+\mathrm{met}\_2$$

With the addition of this new constraint the formal ME-model optimization problem becomes the following:

$$\underset{v,\mu }{\max}\mu$$

s.t.Sv=0

$$v_{\mathrm{formation},\mathrm{Ribosome}}-\sum_{i\in Peptide}\left(\frac{l_{p,i}}{c_{\mathrm{ribo}}\kappa_{\tau }}(\mu +r_{0}\kappa_{\tau })\cdot v_{\mathrm{translation},i}\right)=0$$

$$v_{\mathrm{formation},\mathrm{RNAP}}-\sum_{i\in TU}\left(\frac{l_{\mathrm{TU},i}}{3c_{\mathrm{ribo}}\kappa_{\tau }}(\mu +r_{0}\kappa_{\tau })\cdot v_{\mathrm{transcription},i}\right)=0$$

$$v_{\mathrm{formation},j}-\sum_{i\in \mathrm{generic}\_\mathrm{tRN}A_{\mathrm{AA}}}\left((1+\frac{\mu }{k_{eff,tRNA}})\frac{\mu }{k_{eff,charging}}v_{\mathrm{charging},i}\right)=0,\;\forall j\in Synthetase$$

$$v_{\mathrm{formation},j}-\sum_{i\in \mathrm{enzymatic}\;\mathrm{reaction}}\left(\frac{\mu }{k^{\mathrm{ef}f_{\mathrm{ij}}}}v_{\mathrm{usage},i}\right)=0,\;\forall j\in Enzyme$$

$$v_{\mathrm{formation},j}-\sum_{i\in \mathrm{tRNA}\;\mathrm{anticodons}}\frac{\left(\mu +\kappa_{\tau }r_{0}\right)}{\kappa_{\tau }c_{tRNA,j}}v_{charging,i}=0,\;\forall j\in tRNA$$

$$v_{\mathrm{degredation},j}-\frac{k_{deg,j}}{3\kappa_{\tau }c_{mRNA}}\cdot \frac{\mu +\kappa_{\tau }r_{0}}{\mu }v_{translation,j}=0,\;\forall j\in mRNA$$

$$v_{\mathrm{formation},j}-\frac{\left(\mu +\kappa_{\tau }r_{0}\right)}{3\kappa_{\tau }c_{mRNA}}v_{translation,j}=0,\;\forall j\in mRNA$$

$$v_{\mathrm{formation},j}-\sum_{i\in \mathrm{coenzyme}\;\mathrm{charging}\;\mathrm{reaction}}\left(\frac{\mu }{k^{{\mathrm{activity}}_{\mathrm{ij}}}}v_{\mathrm{usage},i}\right)=0,\;\forall j\in Coenzyme$$

$$v^{L}\leq v\leq v^{U}$$

$$\mu \leq v_{biomass\_dilution}\leq \mu$$

For more details and discussion on the ME-model formulation and theory please refer to [18].

For this study, we were interested in the relative activity of these coenzymes across varying growth conditions. It was thus important that the computed coenzyme abundances were within a reasonable range (**Fig 2**), but quantitative accuracy of the abundance predictions was not necessary. Therefore, accounting for the complex kinetics of the coenzymes throughout the reconstruction was outside the scope of this work. This simple approach effectively relates the rate of coenzyme biosynthesis with its metabolic activity and growth rate.

Changes and corrections were applied to *i*JL1678b-ME to allow the model to be used for this study. First, the biomass constituent demand reaction flux was set to zero. This reaction is included in the default ME-model to account for the synthesis of many of the coenzymes whose activity is modeled directly with the modified ME-model. It is thus no longer necessary. Furthermore, the uptake of any metal cation not included in M9 media was constrained to zero. Malate oxidase was made irreversible as suggested in Monk *et al*. [25]. Corrections were further made to *i*JL1678b-ME to more accurately compute prosthetic group use. For example, acetolactate synthase (ilvH and ilvI) and 2-oxoglutarate dehydrogenase (sucA, sucB, and lpd) were updated to correctly require FAD and thiamine diphosphate as prosthetic groups.

### ME-model parameterization and optimization procedure

The k_eff_ coupling parameters [18] for each metabolic reaction in *i*JL1678b-ME were determined based on a machine learning approach that incorporated enzyme features, network properties, and proteomics data to predict k_eff_s [27,61] from a set of *in vivo* derived enzyme turnover rates [62]. In order to capture the high catalytic efficiency and encourage model activity of pyruvate dehydrogenase the k_eff_ of this reaction was set to 1500 s^-1^ [63]. The remaining k_eff_s for expression machinery and transport reactions were set to a default value of 65 s^-1^ [18], as parameterizing these processes were out of the scope of the machine learning approach. The unmodeled protein value [18] was set to 0 for all simulations. Setting this parameter to 0 allows analysis of poor nutrient sources but results in a computed growth rate in aerobic glucose M9 media of 1.75 hr ^-1^ (**Fig A in S1 Text**), well above the observed growth rate of *E*. *coli*. However, since this parameter is similarly applied to all simulations, the high computed growth rates will not impact the relative changes in computed proteome compositions seen in the various growth conditions. All remaining parameters were set to their default values [23].

Due to non-linearities stemming from the enzyme coupling constraints, ME-models cannot be optimized directly as an LP and thus are solved using a binary search algorithm. To perform the binary search, the following procedure was implemented. First, each symbolic coefficient (growth rate, μ) or reaction bound was compiled into a function by sympy [64]. Then, an LP file was created for the qMINOS solver with all of these symbolic functions evaluated to 0. While the model will always be feasible at 0, starting with a known feasible point results in a basis which can be used to speed up the next run. Afterwards, for each instance of the binary search in μ, values in the LP were replaced by recomputed ones, and the problem was resolved using the last feasible basis. This approach was continued until the difference in maximum feasible μ and minimum infeasible μ was within the defined tolerance (1x10^-13^).

### Computing biomass constituent demand

The amino acid biosynthetic demand was determined based on the translation flux and amino acid composition of each protein in the model. Prosthetic group demand was determined based on the sum of the complex formation fluxes of all enzyme complexes containing the appropriate prosthetic group. Due to the high participation of coenzymes throughout the metabolic network, the biosynthetic flux of each cofactor was determined based on the activity of a reaction in its biosynthetic pathway (**Table A in S1 Text**). This was sufficient given that each cofactor contains one direct biosynthetic pathway. For the comparison of the ME-model predictions to the *i*JO1366 biomass objective function, the computed biomass constituent demand values were normalized by the computed growth rate. For the analysis of conditions dependent biomass compositions, however, the computed biomass demands were normalized by the total protein biomass of the simulation, provided by the “protein_biomass_to_biomass” reaction in the ME-model solution. Global RNA and protein abundances in the ME-model are governed by an empirical RNA-to-protein ratio constraint that is a nonlinear function of growth rate [18]. Since amino acid and prosthetic group abundances will be highly correlated with total protein content, this normalization by protein biomass is required.

## Supporting information

S1 Text Supplemental tables and figures. **Table A in S1 Text**. Mapping of coenzyme to the reaction used to compute its biosynthesis demand. **Table B in S1 Text**. Mapping of amino acid and cofactor name to BiGG ID used in the *E*. *coli* ME-model. **Table C in S1 Text**. Reactions knocked out to produce each ME-model auxotroph. **Fig A in S1 Text**. The computed growth rates for all growth-supporting nutrients, by nutrient source and aerobicity. **Fig B in S1 Text**. The cofactors that are conditionally required for growth, clustered by growth condition. The orange portions of the heatmap denote growth conditions where the cofactor on the x-axis is required for growth. The blue portions denote conditions where the cofactor is not required. The nutrient sources are shown on the left in red, green, yellow, and black for phosphorus, nitrogen, sulfur, and carbon sources, respectively. The colors are light for anaerobic conditions and dark for aerobic conditions. **Fig C in S1 Text.** Change in the total sum of protein, by mass, allocated to each metabolic subsystems when an excess of the essential nutrient listed in the legend is provided. The log_2_ fold change in growth-normalized protein allocation relative to the wild-type model is shown. A subsystem was included if at least one auxotroph saw a log_2_ fold change with an absolute value greater than 0.2. **Fig D in S1 Text.** ME-model computed growth rates of *E*. *coli* auxotrophs in conditions of nutrient limitation. **Fig E in S1 Text**. Principal component analysis of metabolic flux predictions from excess to 10% of the optimal availability of the amino acids and cofactors in **Fig D**. The points corresponding to the metabolite shown above the plot are highlighted in red, and the point size corresponds to the fraction of the optimal availability (large points represent high availability and vice versa). **Fig F in S1 Text**. Model-predicted metabolic changes in response to niacin limitation. **Top panel:** Fraction of protein allocated to each metabolic subsystem by mass for varying niacin availability (columns) **Bottom panel:** Heatmap of reaction fluxes normalized by the maximum flux value for the reaction across all levels of niacin limitation. The 20 reactions with the highest standard deviation are shown and are highlighted in red if the reaction relies on NAD or NADP activity. If reaction fluxes were perfectly correlated throughout the niacin limitation simulations, then these reactions were grouped together. The number in parenthesis shows the number of other reactions represented by the row. The rightmost column depicts a simulation with the highest niacin availability and the leftmost column depicts a simulation with the lowest niacin availability. **Fig G in S1 Text**: Comparison of the computed metabolic flux state in optimal niacin availability and the flux state with 10% of the optimal niacin availability. Reactions are shown in green or red if they are upregulated or downregulated in limited niacin availability, respectively.
(DOCX)

S1 Table Computed growth rates for all growth conditions. (XLSX)

S2 Table Hierarchical clustering summary. The growth conditions represented in each cluster along with the average biomass composition (in mmol / g_protein_).
(XLSX)
