# Supplementary material for: Computation of condition-dependent proteome allocation reveals variability in the macro and micro nutrient requirements for growth
Source: PLoS Comput Biol. 2021 Jun 23;17(6):e1007817. doi: 10.1371/journal.pcbi.1007817 (PMC8259983; doi:10.1371/journal.pcbi.1007817)
Supplement: S1 Text — Table A in S1 Text. Mapping of coenzyme to the reaction used to compute its biosynthesis demand. Table B in S1 Text. Mapping of amino acid and cofactor name to BiGG ID used in the E. coli ME-model. Table C in S1 Text. Reactions knocked out to produce each ME-model auxotroph. Fig A in S1 Text. The computed growth rates for all growth-supporting nutrients, by nutrient source and aerobicity. Fig B in S1 Text. The cofactors that are conditionally required for growth, clustered by growth condition. The orange portions of the heatmap denote growth conditions where the cofactor on the x-axis is required for growth. The blue portions denote conditions where the cofactor is not required. The nutrient sources are shown on the left in red, green, yellow, and black for phosphorus, nitrogen, sulfur, and carbon sources, respectively. The colors are light for anaerobic conditions and dark for aerobic conditions. Fig C in S1 Text. Change in the total sum of protein, by mass, allocated to each metabolic subsystems when an excess of the essential nutrient listed in the legend is provided. The log2 fold change in growth-normalized protein allocation relative to the wild-type model is shown. A subsystem was included if at least one auxotroph saw a log2 fold change with an absolute value greater than 0.2. Fig D in S1 Text. ME-model computed growth rates of E. coli auxotrophs in conditions of nutrient limitation. Fig E in S1 Text. Principal component analysis of metabolic flux predictions from excess to 10% of the optimal availability of the amino acids and cofactors in Fig D. The points corresponding to the metabolite shown above the plot are highlighted in red, and the point size corresponds to the fraction of the optimal availability (large points represent high availability and vice versa). Fig F in S1 Text. Model-predicted metabolic changes in response to niacin limitation. Top panel: Fraction of protein allocated to each metabolic subsystem by mass for varying niacin availability ( [file pcbi.1007817.s001.docx]

**Table A**. Mapping of coenzyme to the reaction used to compute its biosynthesis demand

| **Coenzyme** | **Reaction ID** | **Reaction Name** |
| --- | --- | --- |
| **Coenzyme A** | DPCOAK | Dephospho-CoA kinase |
| **Tetrahydrofolate** | DHFS | Dihydrofolate synthase |
| **NADP** | NADK | NAD kinase |
| **NAD** | NADS1 | NAD synthase (nh3) |
| **Riboflavin** | RBFSb | Riboflavin synthase |
| **Ubiquinone-8** | DMQMT | 3-Dimethylubiquinonol 3-methyltransferase |
| **2-Demethyl-menaquinone 8** | DHNAOT4 | 1,4-dihydroxy-2-naphthoate octaprenyltransferase |
| **Menaquinone 8** | AMMQLT8 | S-adenosylmethione:2-demthylmenaquinole methyltransferase (menaquinone 8) |

**Table B**. Mapping of amino acid and cofactor name to BiGG ID used in the *E. coli* ME-model

| **Cofactor** | | **Amino Acid** | |
| --- | --- | --- | --- |
| **Name** | **BiGG ID** | **Name** | **BjGG ID** |
| 2-Demethyl- menaquinone 8 | 2dmmq8 | L-Alanine | ala__L |
| 2Fe-2S | 2fe2s | L-Arginine | arg__L |
| 4Fe-4S | 4fe4s | L-Asparagine | asn__L |
| Adenosyl- cobalamin | adocbl | L-Aspartate | asp__L |
| bis-MGD | bmocogdp | L-Cysteine | cys__L |
| Biotin | btn | L-Glutamine | gln__L |
| Coenzyme A | coa | L-Glutamate | glu__L |
| Heme O | hemeO | Glycine | gly |
| Menaquinone 8 | mqn8 | L-Histidine | his__L |
| Niacin | nac | L-Isoleucine | ile__L |
| NAD | nad | L-Leucine | leu__L |
| NADP | nadp | L-Methionine | met__L |
| Protoheme | pheme | L-Phenylalanine | phe__L |
| Pantothenate | pnto__R | L-Proline | pro__L |
| Pyridoxal 5'-phosphate | pydx5p | L-Serine | ser__L |
| Pyridoxine | pydxn | L-Threonine | thr__L |
| Ubiquinone-8 | q8 | L-Tryptophan | trp__L |
| Riboflavin | ribflv | L-Tyrosine | tyr__L |
| Siroheme | sheme | L-Valine | val__L |
| Tetrahydrofolate | thf |  |  |
| Thiamine diphosphate | thmpp |  |  |
| Thiamin | thm |  |  |


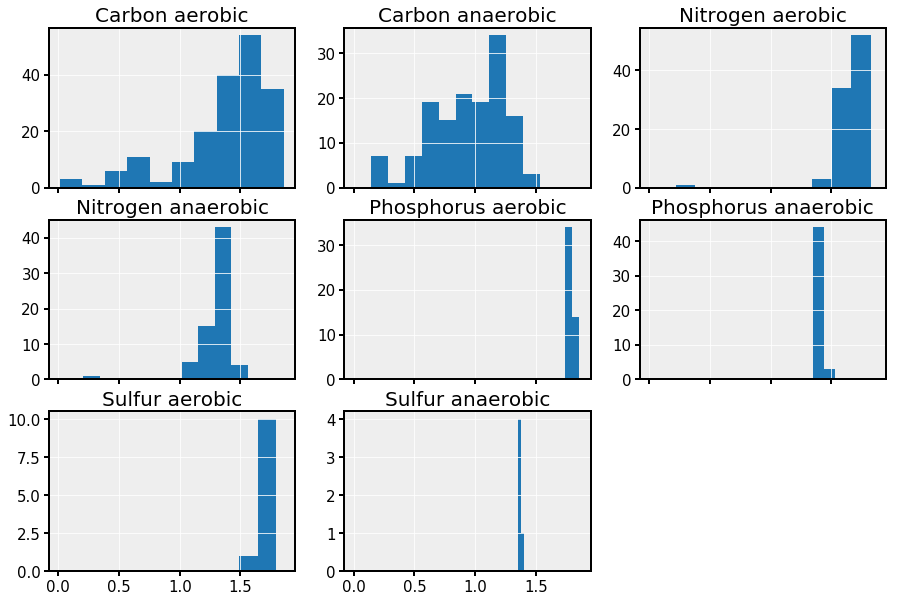


**Fig A**. The computed growth rates for all growth-supporting nutrients, by nutrient source and aerobicity.


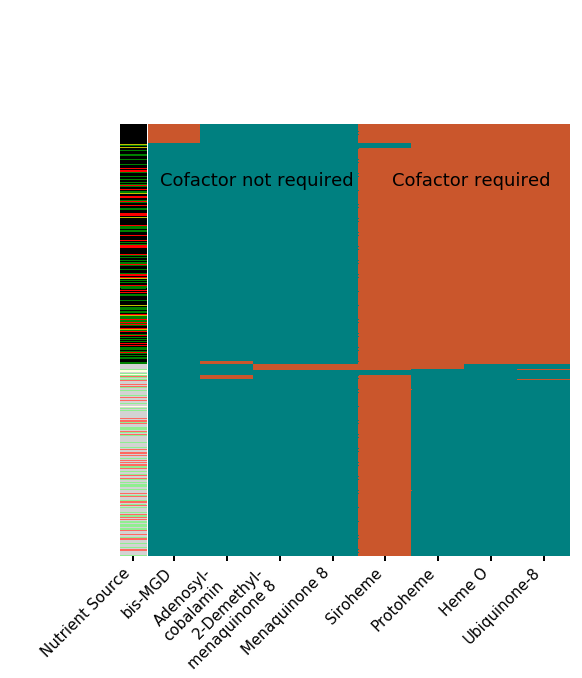


**Fig B**. The cofactors that are conditionally required for growth, clustered by growth condition. The orange portions of the heatmap denote growth conditions where the cofactor on the x-axis is required for growth. The blue portions denote conditions where the cofactor is not required. The nutrient sources are shown on the left in red, green, yellow, and black for phosphorus, nitrogen, sulfur, and carbon sources, respectively. The colors are light for anaerobic conditions and dark for aerobic conditions.

**
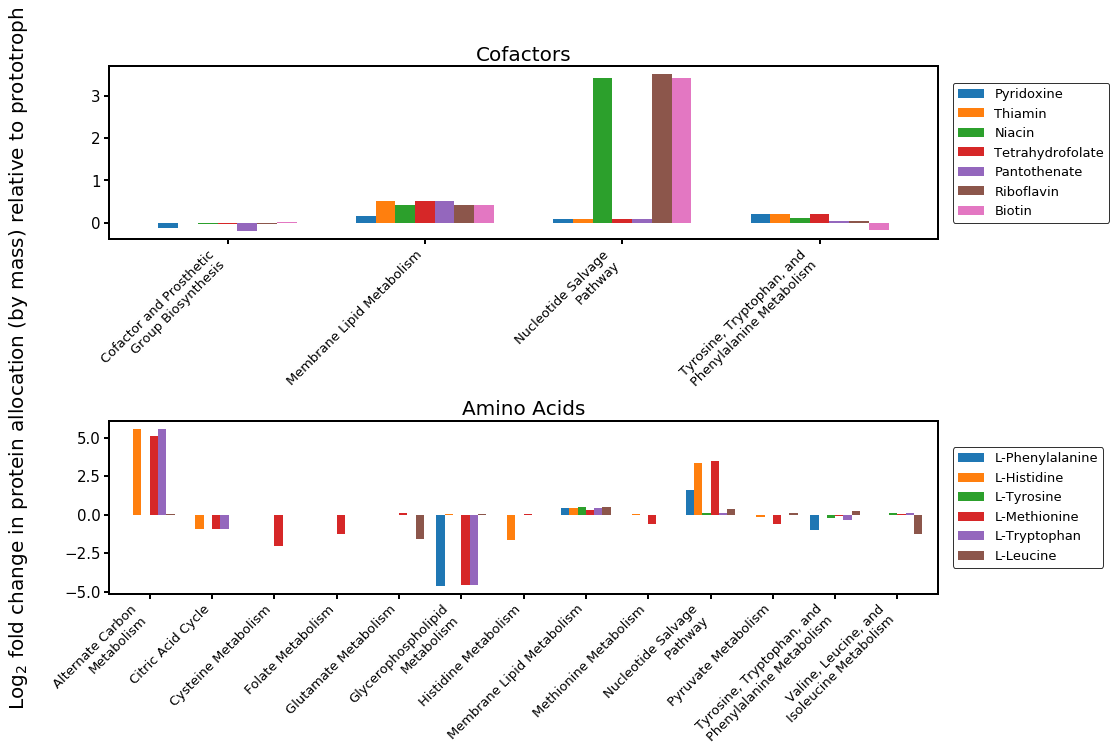
**

**Fig C.** Change in the total sum of protein, by mass, allocated to each metabolic subsystems when an excess of the essential nutrient listed in the legend is provided. The log_2_ fold change in growth-normalized protein allocation relative to the wild-type model is shown. A subsystem was included if at least one auxotroph saw a log_2_ fold change with an absolute value greater than 0.2.

**Table C**. Reactions knocked out to produce each ME-model auxotroph.

| **Micronutrient (BiGG ID)** | **ME-model reactions knocked out for auxotroph** |
| --- | --- |
| Biotin (btn) | ALLTN and DBTS |
| Niacin (nac) | ASPO3 and ASPO4 and ASPO5 and ASPO6 |
| Pantothenate (coa) | PANTS |
| Pyridoxine (pydx5p) | PDX5PS1 and PDX5PS2 |
| Riboflavin (ribflv) | RBFSb |
| Tetrahydrofolate (thf) | DHFR |
| Thiamin (thmpp) | THZPSN31 |
| L-histidine (his__L) | HISTD |
| L-leucine (leu__L) | IPMD |
| L-methionine (met__L) | HSST |
| L-phenylalanine (phe__L) | PPNDH |
| L-tryptophan (trp__L) | IGPS |
| L-tyrosine (tyr__L) | PPND |

**
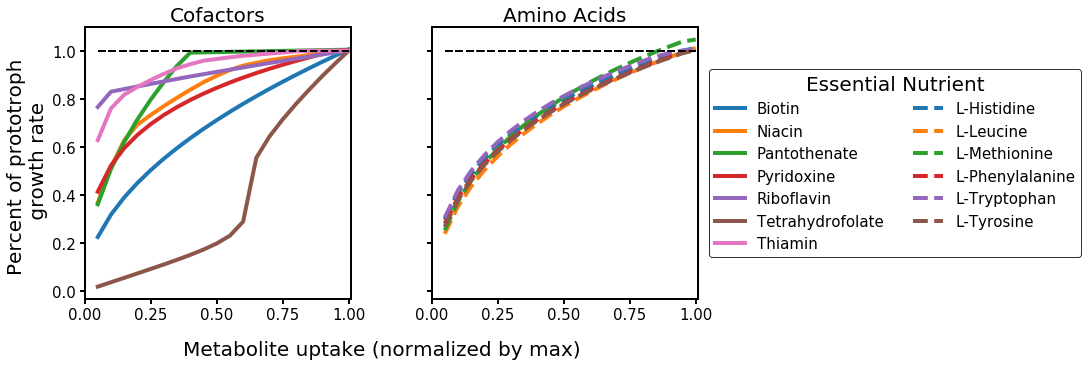
**

**Fig D.** ME-model computed growth rates of *E. coli* auxotrophs in conditions of nutrient limitation.


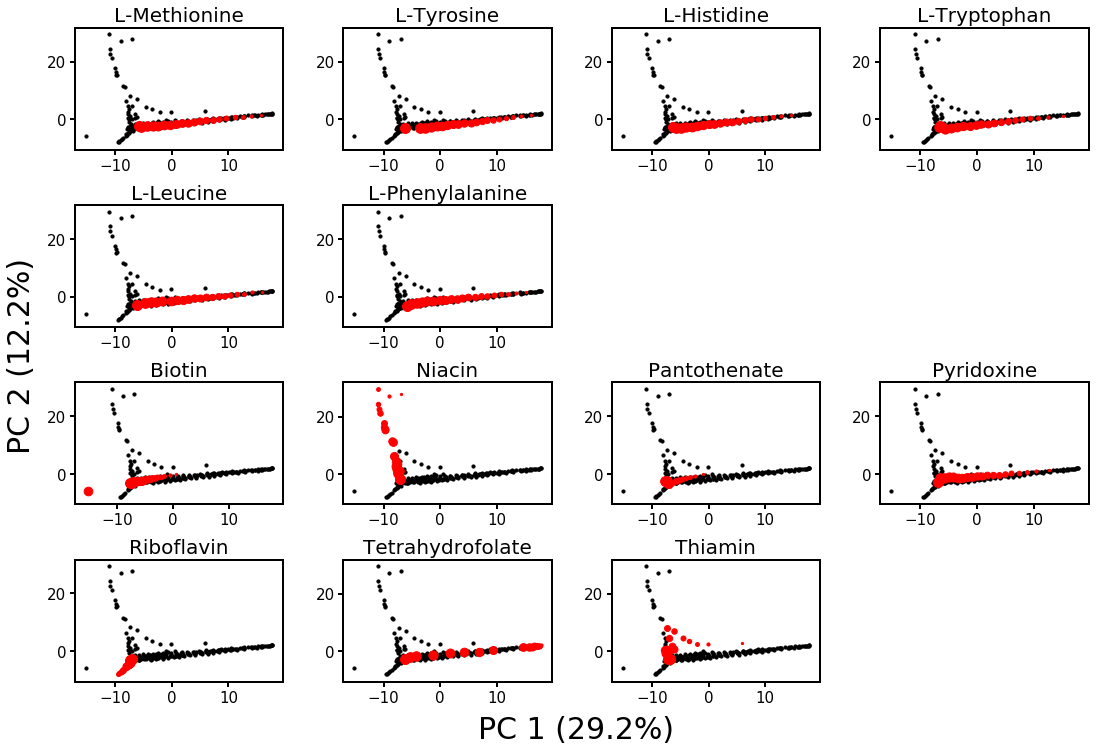


**Fig E**. Principal component analysis of metabolic flux predictions from excess to 10% of the optimal availability of the amino acids and cofactors in **Fig D**. The points corresponding to the metabolite shown above the plot are highlighted in red, and the point size corresponds to the fraction of the optimal availability (large points represent high availability and vice versa).


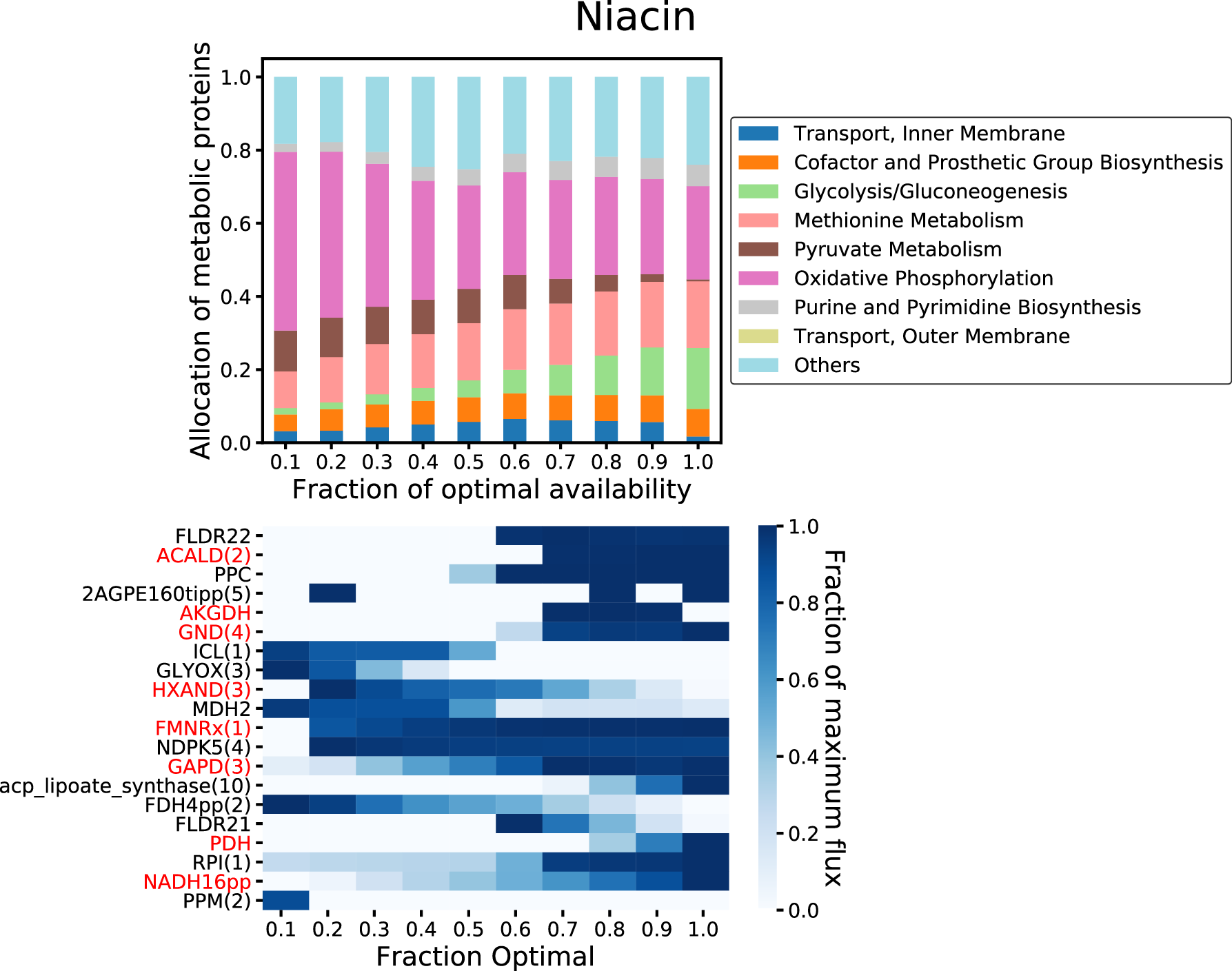


**Fig F**. Model-predicted metabolic changes in response to niacin limitation. **Top panel:** Fraction of protein allocated to each metabolic subsystem by mass for varying niacin availability (columns) **Bottom panel:** Heatmap of reaction fluxes normalized by the maximum flux value for the reaction across all levels of niacin limitation. The 20 reactions with the highest standard deviation are shown and are highlighted in red if the reaction relies on NAD or NADP activity. If reaction fluxes were perfectly correlated throughout the niacin limitation simulations, then these reactions were grouped together. The number in parenthesis shows the number of other reactions represented by the row. The rightmost column depicts a simulation with the highest niacin availability and the leftmost column depicts a simulation with the lowest niacin availability.

**
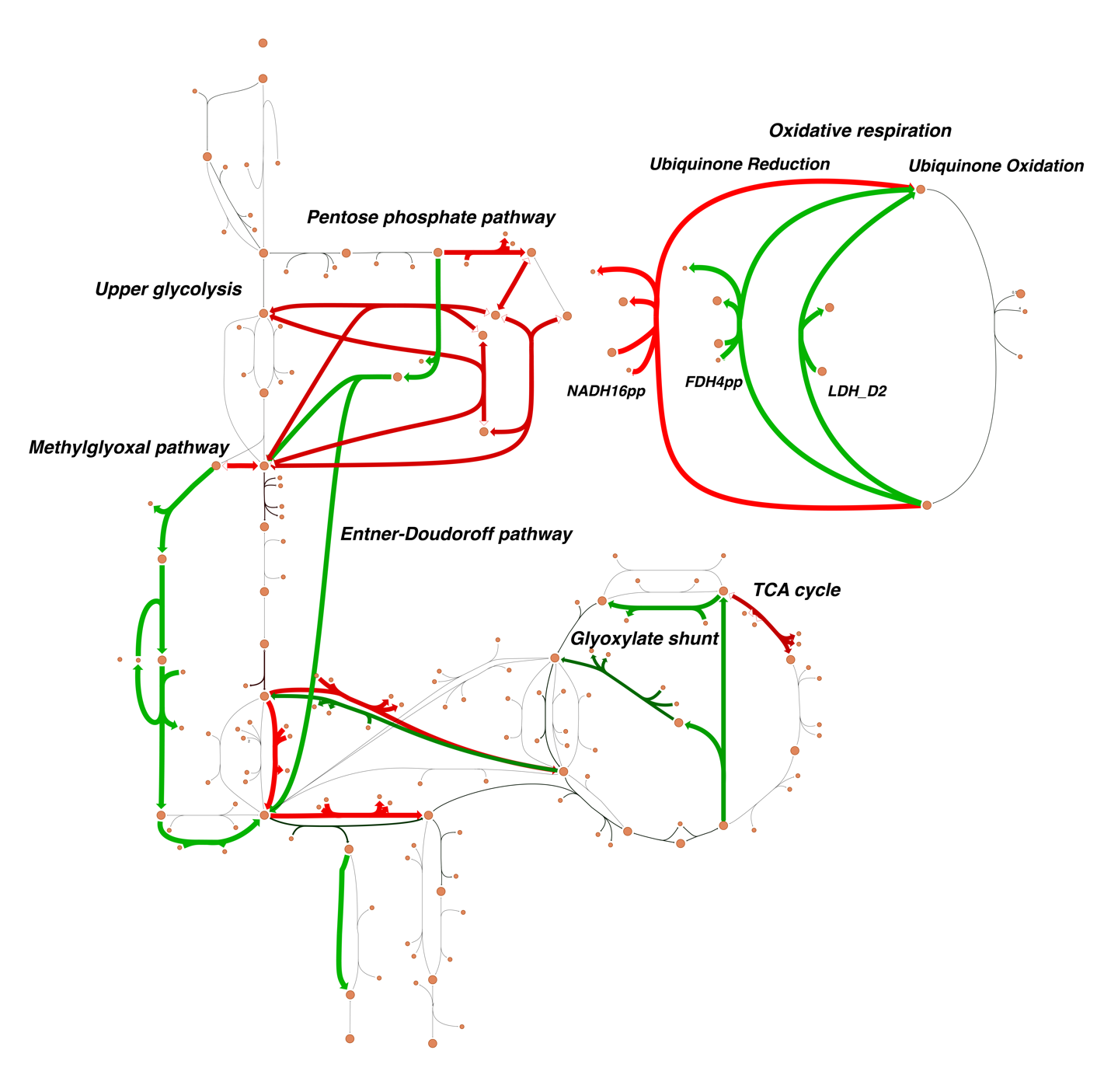
Fig G**: Comparison of the computed metabolic flux state in optimal niacin availability and the flux state with 10% of the optimal niacin availability. Reactions are shown in green or red if they are upregulated or downregulated in limited niacin availability, respectively.
